# What do Web-Use Skill Differences Imply for Online Health Information Searches?

**DOI:** 10.2196/jmir.2051

**Published:** 2012-06-13

**Authors:** Markus A Feufel, S Frederica Stahl

**Affiliations:** ^1^Harding Center for Risk LiteracyCenter for Adaptive Behavior and CognitionMax Planck Institute for Human DevelopmentBerlinGermany

**Keywords:** Public access to information, computer literacy, information-seeking behavior, patient education handout, human factors, digital divide, information retrieval

## Abstract

**Background:**

Online health information is of variable and often low scientific quality. In particular, elderly less-educated populations are said to struggle in accessing quality online information (digital divide). Little is known about (1) how their online behavior differs from that of younger, more-educated, and more-frequent Web users, and (2) how the older population may be supported in accessing good-quality online health information.

**Objective:**

To specify the digital divide between skilled and less-skilled Web users, we assessed qualitative differences in technical skills, cognitive strategies, and attitudes toward online health information. Based on these findings, we identified educational and technological interventions to help Web users find and access good-quality online health information.

**Methods:**

We asked 22 native German-speaking adults to search for health information online. The skilled cohort consisted of 10 participants who were younger than 30 years of age, had a higher level of education, and were more experienced using the Web than 12 participants in the less-skilled cohort, who were at least 50 years of age. We observed online health information searches to specify differences in technical skills and analyzed concurrent verbal protocols to identify health information seekers’ cognitive strategies and attitudes.

**Results:**

Our main findings relate to (1) attitudes: health information seekers in both cohorts doubted the quality of information retrieved online; among poorly skilled seekers, this was mainly because they doubted their skills to navigate vast amounts of information; once a website was accessed, quality concerns disappeared in both cohorts, (2) technical skills: skilled Web users effectively filtered information according to search intentions and data sources; less-skilled users were easily distracted by unrelated information, and (3) cognitive strategies: skilled Web users searched to inform themselves; less-skilled users searched to confirm their health-related opinions such as “vaccinations are harmful.” Independent of Web-use skills, most participants stopped a search once they had found the first piece of evidence satisfying search intentions, rather than according to quality criteria.

**Conclusions:**

Findings related to Web-use skills differences suggest two classes of interventions to facilitate access to good-quality online health information. Challenges related to findings (1) and (2) should be remedied by improving people’s basic Web-use skills. In particular, Web users should be taught how to avoid information overload by generating specific search terms and to avoid low-quality information by requesting results from trusted websites only. Problems related to finding (3) may be remedied by visually labeling search engine results according to quality criteria.

## Introduction

Unlike most information environments in human history, the Internet provides almost unlimited and constantly changing information sources of varying and often unknown quality. These properties pose a challenge to people navigating the Internet, in particular if information contents are highly specialized as in the medical domain. Analyses of online sources show that online health information is of variable and in most cases low scientific quality [1,2]. In addition, studies suggest that a digital divide limits older and less-educated people’s Web-use skills and, ultimately, their access to online information [3]. Cognizant of these challenges, researchers and both governmental and nongovernmental institutions have attempted to objectively map the quality landscape of the Internet [4-6]. These initiatives provide site recommendations and transparency criteria that can be used to evaluate websites, including information about data sources, authorship, or last update. Although health information seekers may be aware of such criteria, only “few...notice and later [remember] from which websites they retrieved information or who stood behind the sites” (page 576 [7]). Instead, Web users rely on and trust the first few results provided by widely used search engines, such as Google, Bing, or Yahoo! [7,8].

Apart from a general consensus that people are poor at searching and evaluating online information [9], little is known about how user characteristics, such as Web-use skill differences, affect online behaviors and may inform interventions to facilitate retrieval of good-quality information [10,11]. Moreover, most research into online behavior is limited to mock-up sites [12], restricted search spaces [13,14], and quantifiable outcome measures such as search time, number of search terms entered, or clicks [15]. To complement experimental research and generate first hypotheses about how to help health information seekers find good-quality information, we identified qualitative differences in skilled versus less-skilled Web users’ (1) attitudes toward online health information, (2) technical skills, and (3) cognitive strategies for search and evaluation of online health information.

## Methods

### Participants

Participants were native German speakers recruited from the participant pool at the Max Planck Institute for Human Development in Berlin, Germany. To identify meaningful differences in participants’ Web-use skills, we used demographic factors that have been shown to facilitate access and use of online information, including younger age [16] and higher education [17]. In addition, we used a Web-oriented digital literacy survey measure as a validated proxy for people’s actual Web-use skills [18,19]. Finally, we characterized cohorts in terms of online behavior using items from the General Social Survey (GSS) [10], which provided a quantitative measure of people’s health information-seeking behavior, offline and online, as well as information about Web access and overall time spent online.

### Cohort Characteristics

The older cohort consisted of 12 individuals (6 women) who were at least 50 years of age (mean 65, SD 4.3 years). Most members of this cohort (9/12, 75%) were secondary school (*Realschule*) graduates; 3 participants had a university degree. The younger cohort consisted of 10 individuals (5 women) who were all younger than 30 years of age (mean 23, SD 3.3 years) and on average more highly educated than the less-skilled cohort. Of the 10 participants in this group, 9 had graduated from high school (*Gymnasium*) and 5 were pursuing a university degree.

Concerning health information-seeking behavior, all participants reported having searched for health information at least once in the previous year. To do so, all but 3 of the older participants (19/22, 86%) stated that they had searched online. Apart from these similarities, members of the younger cohort reported spending almost 6 times more time online (mean 1022, SD 773 minutes/week) than their older counterparts (mean 175, SD 120 minutes/week). We also found strong cohort differences in participants’ Web-oriented digital literacy scores. Whereas older participants obtained on average about half of 130 possible literacy points (mean 63, SD 22 points), members of the younger cohort obtained about three-quarters of the possible points (mean 98, SD 21 points). Thus, the demographic cohort differences of age and education level as well as the Web-related differences of time spent online and digital literacy scores spoke to a general advantage of the younger cohort in terms of accessing and using online information [16,17]. We therefore refer to these cohorts as skilled and less-skilled Web users.

### Materials

Most search tasks used to study online health information-seeking behavior have asked participants to search for facts, as in “What is the definition of being overweight?” [7,8]. However, prior research assumed [13,20,21] and “a great many health seekers say the resources they find on the Web have a direct effect on the decisions they make...and on their interactions with doctors” (page 3 [22]). Thus, we provided search scenarios that asked participants to search for facts, *as well as *inference scenarios that asked them to make decisions or recommendations based on information retrieved online (see Table 1).

We designed 3 search scenarios to capture the most common health facts searched online [23]: information about diseases and symptoms (searched by 66% of US adult Web users), information about prescription drugs (45%), and information about medical tests (16%). Similarly, we developed 4 inference scenarios capturing common motivations for inference-related online searches [24]. One inference scenario related to pregnancy (searched by 19% of US adult Web users) and 2 scenarios concerned vaccination decisions (16%). We added a self-treatment and diagnosis scenario because participants frequently commented on the phenomenon during the first 8 sessions and “there is great concern in the medical establishment that e-patients are self-diagnosing and self-medicating” (page 15 [23]). Surveys suggest that 18% of US adults use the Internet to self-diagnose [23].

**Table 1:** Search scenarios presented to participants.

Scenarios		Common search motivations identified in the literature^a^
**Search scenarios**		
	A physician talked to you/your husband/your father about taking a PSA^b ^test. The doctor explained that the validity of the test has been questioned by a number of physicians. How do you assess the validity of the test?	16% search for interpretations of medical test results [20]
	Your doctor prescribes eszopiclone and lets you know that you might develop a bad aftertaste when taking the medication. You do not develop a bad aftertaste, but feel occasionally nauseous. Could nausea be a result of taking eszopiclone?	45% search for information on prescription drugs [20]
	Your partner/parent has been told by his/her physician that he/she has an increased risk of a stroke. How would you recognize a stroke?	66% search for a particular illness or condition [20]
**Inference scenarios**		
	Your daughter’s/your sister’s gynecologist recommends that she should get the gardasil vaccination. What would you advise her to do?	16% search for immunization- and vaccine-related information [21]
	Close friends of yours are contacted by their pediatrician, who recommends that their child get an MMR^c ^vaccination. Your friends discuss the matter with you. Should their child be vaccinated?	16% search for immunization- and vaccine-related information [21]
	Your pregnant daughter’s/friend’s gynecologist suggests that she might want to undergo an amniocentesis. What would you advise her?	19% search for pregnancy-related information [21]
	You wake up one morning with a swollen elbow. What could that be? How would you go about treating it?	18% state using the Internet to self-diagnose [20]

^a ^Search motivations are based on survey results provided by the Pew Internet & American Life Project. Percentages refer to representative samples of US Web users.

^b ^Prostate-specific antigen.

^c ^Measles-mumps-rubella.

Given that most of the scenarios target particular populations, we asked participants to search for information for friends or family members belonging to these populations. Research suggests that 81% of adult Web users search online information for others [23], so this change of perspective should not have reduced the perceived relevance of the scenarios. All study materials including consent forms were approved by the ethics committee at the Max Planck Institute for Human Development.

### Data Collection and Procedure

Sessions lasted about 90 minutes and were conducted by one of the authors (FS) who observed participants throughout the sessions. To be as unobtrusive as possible, we explained the think-aloud method [25] to participants at the beginning of the sessions, asking them to verbalize thoughts during online searches. In case participants discontinued thinking aloud, a list of probing questions was devised. Examples include “How did you come up with your search term?”, “Why did you click on this link?”, and “What are you looking for on this site?” to probe for search implementation, site selection, and site navigation strategies, respectively. To record verbal protocols and on-screen behaviors, we used a MacPro laptop (Apple Inc, Cupertino, CA, USA) with iShowU screen-capture software (shinywhitebox ltd, Wellington, New Zealand).

After providing informed consent, participants were given one of the scenarios chosen at random. Without specifically requesting an Internet search, we asked them to report on how they would approach the scenario. This part was used to identify attitudes toward online health information and typical health information search patterns. If online search was not part of the strategy they mentioned, they were queried at the end of the session about the role they assign to online health information. After this part, participants were shown the computer and how to open a blank browser window. Then, they were given the remaining 6 scenarios, consecutively and in random order. Participants were unrestricted in terms of search time, how to approach searches, and which websites to visit. After having worked on the scenarios, participants filled out the surveys used to characterize cohorts.

### Data Analysis

Verbal protocols were transcribed immediately after each session. Both authors then independently coded protocols and screen-captured online behaviors. Diverging codes were discussed until consensus was reached. Codes reflected *attitudes *toward online health information, *technical skills *(based on observable on-screen behaviors), and *cognitive strategies *(based on verbal protocols). Attitudes were coded with respect to the *categories of* *information *participants would or would not search for online and their *rationales *for doing or not doing so. Technical skills were subdivided into *search implementation*, which refers to the website(s) accessed to begin the search and the generation of search term(s); *site selection*, which refers to how people decided which of the search results to follow up on in detail; and *site navigation, *which describes how participants traveled through a selected site. Following decision-making research [26], we subdivided cognitive strategies into *search intentions *used to structure information retrieval; *rules for information evaluation*, referring to how participants interpreted online information and modified their search; *stopping rules*, defined by how participants chose to end their search; and *inference rules*, defined by how they integrated the retrieved information into a decision or recommendation.

## Results

In the following, we describe the impact of Web-use skill differences on (1) user attitudes, (2) technical skills, and (3) cognitive search and evaluation strategies during online health information searches. Table 2 summarizes our findings, which we discuss in turn.

**Table 2:** Summary of results.

Outcome	General findings	Skill differences	
		Less-skilled cohort	More-skilled cohort
**Attitudes**			
	Participants hesitated to use online health data (20/22, 91%), in particular to make inferences	Rationale: the plethora of data cannot be managed (6/12, 50%)	Rationale: the quality of online data is low (8/10, 80%)
	Although data quality was a matter of concern...	...keywords may override distrust toward sites (6/12, 50%)	...once accessed, data from any site were used (4/10, 40%)
**Technical skills**			
Search implementation	Reliance on search engine Google.de (22/22, 100%)	No differences observed	Few entered URLs directly (2/10, 20%)
	Use of search terms (20/22, 91%); often misspelled; rarely corrected	83% used a single search term (10/12); 67% used search term suggestions (8/12); 17% used natural language phrases (2/12)	100% used two or more search terms (10/10)
Site selection	Reliance on first 5 links on first search engine result page (20/22, 91%)	Selection based on keywords (often unrelated to original search) inferred from links/excerpts (7/12, 58%)	Selection based on data sources inferred from URL or links/excerpts (9/10, 90%)
Site navigation	Relevance of website contents was appraised	Text was read rather than scanned. In 23% of searches (14/61) links were followed up.	Text was scanned for keywords. In 6% of searches (3/49), links were followed up.
	Information was rarely cross-referenced	No cross-referencing or use of tabs (0/12, 0%).	Use of multiple tabs to compare results (2/10, 20%).
**Cognitive strategies (based on inference scenarios only)**			
Search intentions	People searched for online contents related to personal, a priori opinions, knowledge, cues, or expert opinions.	Distribution of intentions: 70% a priori opinions (21/30); 10% cues (3/30); 10% knowledge (3/30); 10% expert opinions (3/30)	Distribution of intentions: 14% a priori opinions (4/28); 29% cues (8/28); 29% knowledge (8/28); 29% expert opinions (8/28)
Information evaluation	Information was trusted if consistent with search intentions—that is, if...	...a website confirmed a priori opinions (21/30, 70%) or yielded search contents (9/30, 30%)	...a website confirmed a priori opinions (4/28, 14%) or yielded search contents (24/28, 86%).
Stopping rule	Search was stopped once the first piece of online information satisfied search intentions	No participant further cross-referenced (0/12, 0%)	20% further cross-referenced information (2/10)
Inference rule	Participants were hesitant to make inferences based on online searches, except when they searched to confirm personal, a priori opinions	In 73% of the inference queries, inferences were made based on a priori opinions (22/30). In 27% no inferences were made (8/30).	In 14% of the inference queries, inferences were made based on a priori opinions (4/28); in 7% based on cues (2/28). In 79% no inferences were made (22/28).

### Attitudes Toward Online Health Information Seeking

Participants identified clear boundaries as to when and for what purpose they would access online health information. For instance, all participants who were asked to self-diagnose and treat a swollen elbow (14/22) made statements such as “I don’t trust the Internet when it comes to symptoms...home remedies maybe, but diagnoses definitely not.” In fact, 7 of the 14 participants asked to self-diagnose (50%) refused to search online; the other half searched for “swollen elbow” but, after having scanned the first results, decided that looking up symptoms online was inappropriate and discontinued their search.

Similarly, during the first scenario, when not explicitly asked to use the Internet, 7 of the 12 less-skilled participants (58%) did not consider the Web; only 1 (8%) referred to it as the primary source of health information. Although all 10 skilled participants mentioned the Internet, only half identified it as their primary source. In general, 20 of 22 participants (91%) made statements reflecting distrust toward online sources: “First I would turn to my doctor; then to family members, maybe close friends. A distant third would be online research...a very distant third” or “My trust in the Internet is negatively correlated to the importance I ascribe to an issue: the more important the issue, the less I trust, and thus rely on, the Internet.”

When we asked participants to actually surf the Web (after completing the first scenario), verbal protocols suggested different reasons for why more- versus less-skilled Web users were hesitant to access online health information. Half of the less-skilled users (6/12) made statements such as “Online one gets easily carried away” and “I shouldn’t search online by myself or I will be bombarded by all this information. One has to be a professional to ask the right questions.” Thus, less-skilled participants expressed concern about the amount of available information and their lack of navigation skills. In contrast, 8 of the 10 skilled participants (80%) made statements such as “It’s difficult to find valid health information online...anyone can put a site online.” A total of 5 participants (50%) stated that health was too important a topic to rely solely on online resources: “You can’t always trust Google. I’d rather ask a doctor or pharmacist.” All 10 skilled Web users (100%) questioned the quality of and trust they had in online sources at least once during their online searches.

The hesitant attitude toward online health information seems at odds with earlier studies, which assumed that online information influences medical decision making [13,20-22]. This is only an apparent contradiction, however. Once participants accessed a website in our study, their concerns about data quality vanished. Among the 12 less-skilled users, only 2 (17%) mentioned quality issues explicitly once online, and 6 of them (50%) doubted a source but, after identifying an interesting keyword, clicked on the link and reasoned about the content of the site nonetheless. Similarly, skilled participants only infrequently referred to trust once they accessed a page. In 4 of their 20 searches, 4 participants read and considered information in their reasoning, even though they had expressed distrust of the source before deciding to visit the website. Thus, although people voice concerns about data quality issues, once they access a website, even skilled Web users are preoccupied with processing website contents.

### Technical Skills Based on Observed Online Behaviors

Overall, participants performed 110 online searches: 52 (47%) were related to search scenarios and 58 (53%) were related to inference scenarios (see Table 3). Prior observational research has found that people may search from 5 to 20 minutes before they stop [7,8]. Our results suggest search times of about 5 minutes for search scenarios and 6 minutes for inference scenarios. In general, more-skilled participants spent about 2 minutes less time searching than their less-skilled counterparts.

**Table 3:** Number of online searches and mean search time by search scenario and cohort type.^a^

Scenario type		Number of online searches			Mean search time (minutes:seconds)		
		Less-skilled cohort	More-skilled cohort	Total	Less-skilled cohort	More-skilled cohort	Total
Search scenarios		31	21	52	5:04	4:17	5:16
	Validity of PSA^b ^test	10	9	19	6:32	4:31	5:48
	Drug side effects	11	9	20	6:25	4:37	5:31
	Stroke symptoms	10	3	13	3:50	2:34	3:35
Inference scenarios		30	28	58	7:53	4:57	6:22
	MMR^c ^vaccination	12	9	21	9:03	6:00	7:45
	Gardasil vaccination	8	9	17	8:21	5:13	6:29
	Amniocentesis	10	10	20	6:04	3:46	4:49
	Self-diagnosis^d^	(3)	(4)	(7)	(2:15)	(2:34)	(2:27)
Overall		61	49	110	6:45	4:40	5:47

^a ^Given that the first scenario was not followed up with actual online searches, there is a total of (6 – 1) × 22 = 110 searches.

^b ^Prostate-specific antigen.

^c ^Measles-mumps-rubella.

^d ^In response to the self-diagnosis scenario, 7 searches were started but not completed, which are added in parentheses but not in the totals.

#### Search Implementation

In line with prior observational work [7,8], participants mainly relied on search engines to access online information (in 107/110, 97% of the searches). All 22 participants showed a strong preference for the search engine Google.de. Also echoing earlier findings, 91% (20/22) of the participants searched for health information using single-word search terms, which were often left uncorrected if misspelled**, **even when the search engine provided alternative, correct spellings. This cohort comparison extends prior research by revealing differences with respect to the kinds and specificity of the search terms generated.

Of the 12 less-skilled Web users, 10 (83%) entered single-word search terms, often the disease or medication discussed in the scenario. The remaining 2 (17%) participants entered full natural language phrases, such as “How would I recognize a stroke?” Consequently, the majority of the less-skilled participants received rather general search results. Of the 12 less-skilled participants, 8 (67%) considered the search term options suggested by Google’s search bar to help specify search terms. They did not understand, however, how that would change their search results. For example, 1 participant searched for eszopiclone (a sleeping drug). When Google suggested the search term *side effects *to specify the query, the participant accepted the suggestion but, looking at the results, exclaimed “Oh, look at all those side effects. Every title listed includes side effects as a catchphrase...No, I would definitely talk to my doctor [before taking this medication].” In contrast, all 10 skilled Web users performed Google searches based on two or more single-word search terms. They entered a disease or medication and specified the category of information they were looking for, such as “eszoplicone side effects”, “HPV vaccinations MD opinions”, or “MMR vaccine tolerance.” They mostly ignored search terms suggested by the search engine, indicating prespecified search intentions.

#### Site Selection

About 91% (20/22) of all participants chose sites ranked among the first 5 links on the first result page. Participants left the first page provided by the search engine in only 7 of 107 (7%) Google searches. All of these 7 searches were related to the drug eszopiclone, a type of sleeping pill, available in the United States but not in Germany, so that the websites provided on Google.de were all in English. Thus, all of the 7 searchers leaving the first result page did so in hope of finding a German-language page.

Apart from a focus on the first result page, cohorts selected links differently. More than half of the less-skilled Web users (7/12, 58%) scanned link titles and excerpts provided by Google for keywords that triggered their interest. The remaining 5 (42%) participants occasionally ignored search engine titles and excerpts and identified keywords on websites. The keywords participants identified were often unrelated to their original search term entry. All 12 participants entered a disease as the search term at least once, read about side effects in an excerpt or title, and then decided to visit that site, without having actively searched for side effects.

Of the 10 skilled Web users, 9 (90%) decided which site to follow up by information source rather than keywords. They either directly recognized the source in the case of commonly known websites such as Netdoktor.de or extrapolated the type of source (eg, patient reports, health forums) from link titles, excerpts, or URLs. All members of this cohort relied at least once on Wikipedia.de to obtain a general overview; 6 (60%) of them expressed distrust toward available health forums and frequently chose not to visit a forum, even if it was ranked among the first entries on the results page.

#### Site Navigation

Once they selected a website, all participants started scanning the text to confirm its relatedness to the search topic. Cohorts differed in their scanning strategies. Less-skilled Web users started reading the text and followed the links provided on the websites. In 14 of 61 searches (23%), less-skilled participants accessed more than one page of a chosen website or looked at the table of contents. In addition, they considered information that was unrelated to their initial queries. For example, to find evidence concerning amniocenteses for a pregnant friend, 5 of the 12 (42%) less-skilled participants clicked on Netdoktor.de. On this website, the sidebars listed infomercials about ultrasound tests for pregnant women. Although it was not listed as an alternative to amniocentesis, the 5 participants followed the link and eventually suggested that their friend get an ultrasound as well.

Skilled participants used a more focused strategy to navigate websites and satisfy search intentions. In only 3 of 49 (6%) searches did skilled Web users access more than the first page of a chosen website. In all 3 instances, the original website was Wikipedia. Of the 10 skilled users, 4 (40%) commented that Wikipedia provided a good overview of any given topic and was a good place to start a search. To avoid reading too much text, 4 of the 10 (40%) skilled participants used the shortcut CTRL+F to bring up the Find utility and indentify search-related keywords on the website. To compare website contents, 2 (20%) of the skilled participants used multiple tabs to shuttle between websites.

### Cognitive Strategies Based on Verbal Protocols

#### Search Intentions

There were marked differences in how the cohorts approached inference scenarios (see Table 4). In 21 of 30 (70%) instances, less-skilled participants based their search on a priori *opinions *toward the diseases, medications, or procedures mentioned in the scenarios, such as “I don’t like medications so I wouldn’t take it” or “I’m very skeptical about vaccines.” In the extreme, a participant self-identifying as an anthroposophist exclusively looked up websites about his philosophy of health. This tendency was so strong that for 6 of 30 inference-related queries (20%), less-skilled participants expressed a preference not to search online at all (and started searching only after having been prompted by the researcher) because they had already made up their mind about the scenario. Less-skilled Web users rarely searched for biomedical knowledge (in 3 of 30 queries), decision-relevant cues (3 of 30), or expert opinions (3 of 30).

More-skilled participants, on the other hand, searched to confirm a priori opinions in only 4 of 28 (14%) inference-related queries. In the 3 inference scenarios, members of this cohort generally used a more varied set of search strategies than their less-skilled counterparts (2.1 versus 1.3 different strategies) and more often searched for biomedical *knowledge *of diseases, medications, or procedures (in 8 of 28, 28% of the queries; eg, “Let’s see how this medication works.”); decision-relevant *cues *(8 of 28 queries; eg, “Well, I guess the question is what are the side effects?”); or *expert opinions *by health professionals, governments, or health organizations (8 of 28 queries; eg, “What kind of doctor performs a PSA test? Urologist, right? Let’s look what they say.”). These search intentions echo the search implementation technique observed in this more-skilled cohort. Their search terms first specified the medical condition or procedure and then an information category depending on how they intended to approach the scenario by adding one or more search terms related to biomedical knowledge (eg, “physiology”), inference-relevant cues (eg, “side effects”), or “MD opinions”.

**Table 4:** Participants’ type of online search and evaluation strategy for inference scenarios.^a^

Inference scenario		Strategy type^b^												
		1	2	3	4	5	6	7	8	9	10	11	12	Average
**Less-skilled participants**														
	MMR^c ^vaccine	K	O	O	O	O	O	K	O	O	O	O	EO	
	Gardasil vaccine	O	O	O			O	C	O		O		EO	
	Amniocentesis	K		O	O	O		C	O	O	O	C	EO	
	No. of strategy types	2	1	1	1	1	1	2	1	1	1	2	1	1.3
**More-skilled participants**														
	MMR vaccine	EO	K	EO	C	K		EO	EO	K	K			
	Gardasil vaccine	C	C	EO	O	K	EO	C		O	K			
	Amniocentesis	O	C	K	C	K	C	EO	O	EO	C			
	No. of strategy types	3	2	2	2	1	2	2	2	3	2			2.1

^a ^Strategies in verbal protocols that were not followed up with actual online searches (ie, in response to the first and the self-diagnosis scenarios) are not included in this table.

^b ^O = participants searched to confirm a priori *opinions*, K = participants searched for biomedical *knowledge*, C = participants searched for decision-relevant *cues*, EO = participants searched for *expert opinions *on a topic.

^c ^Measles-mumps-rubella.

#### Information Evaluation

Confirming survey studies, we found that consistency encouraged participants to consider and trust Web content [23]. However, the kind of consistency participants were looking for was not between websites, as previously suggested, but between information retrieved online and search intentions. In 21 of 30 (70%) inference-related queries, less-skilled users evaluated information by relating it to their opinions about health issues. They trusted websites when they found that “my opinion is reflected here” or continued to search because they believed “there must be people out there who don’t believe in vaccines either.” Conflicting information was ascribed to bias on the part of the author(s): “Of course doctors support vaccinations. They make money off them.” Alternatively, participants downplayed information disagreeing with their opinions. For instance, on visiting a site about the side effects of vaccinations, a participant who was generally pro vaccine stated: “I know there is a discussion about this procedure, but life is full of risks.”

Skilled participants searched and evaluated information that concurred with their more varied repertoire of search intentions or strategies. For example, when two sites listed contradicting side effects, 1 participant searched for biomedical explanations to better understand the side effects in question. In a similar instance, another participant changed the cue he considered relevant. On finding two sites describing the same side effect as either infrequent or frequent, the participant stopped evaluating the procedure based on side effects and aimed at identifying whether he really needed the procedure. Interestingly, none of the skilled participants who searched for expert opinions (see Table 4) acknowledged the possibility of conflicting opinions, nor did they continue searching after retrieving a first opinion.

#### Stopping Rule

The stopping rule participants in both cohorts commonly used was closely linked to their search intentions. That is, once they found the first piece of information that satisfied their search intentions (in the case of more-skilled participants) or was consistent with their health-related opinions (in the case of less-skilled participants), participants stopped searching. Only 2 of the more-skilled participants (20%) further cross-referenced such information once they had retrieved it.

#### Inference Rule

As stated above, participants were generally hesitant to rely on the Internet to make decisions or recommendations. However, less-skilled Web users based 73% (22/30) of their recommendations or decisions on a priori opinions, which is reflected in statements such as “Yes, I would get vaccinated; I’m generally in favor of vaccinations.” Among the 10 more-skilled participants, 2 women expressed a priori opinions about the reproduction-related scenario and 2 men were generally in favor of vaccinations. In these 4 out of 28 (14%) instances, participants made recommendations based on their opinions. More-skilled Web users made recommendations based on inference-relevant cues in only 2 of 28 (7%) instances. For example, 1 participant referred to rare side effects as a reason to get vaccinated: “According to Wikipedia I would get vaccinated...the side effects are not that bad after all.” In all other instances, participants reasoned about the online information but did not arrive at a conscious plan of action or recommendation.

## Discussion

This study described the impact of Web-use skill differences on (1) user attitudes, (2) technical skills, and (3) cognitive search and evaluation strategies during online health information searches. After summarizing the results, we will present first hypotheses concerning interventions that, based on our findings, may facilitate the search for good-quality online health information.

First, both more- and less-skilled Web users were hesitant to use the Internet when making medical decisions, although for different reasons. Whereas poorly skilled Web users were concerned about managing data quantity, more-skilled users were concerned about its quality. However, once participants accessed online information, concerns about data quality vanished, independent of skill level. This suggests that, independent of actual Web-use skills, support interventions should focus on Web users’ search implementation and site selection efforts.

Second, in terms of technical skills, skilled Web users effectively implemented and filtered information according to search intentions and data sources (eg, patient blog versus health forum). Less-skilled users had difficulties translating search intentions into search terms. Although they intended to search for relevant keywords on search engine result pages, less-skilled users were easily distracted by interesting but query-unrelated information, therefore often depending on coincidental findings. Again, this suggests that interventions should focus on search implementation and site selection efforts, in particular for users with poor Web-use skills.

Third, both cohorts evaluated search results based on consistency, although in different ways. Whereas most skilled Web users stopped a search once they found the first website providing the contents they were looking for, most less-skilled users posed less-specific queries and trusted information that was consistent with their health beliefs. Neither cohort systematically identified the quality of the retrieved information. This confirms studies suggesting that Web-use skills are necessary but not sufficient to guarantee access to and use of good-quality health information [27].

### Limitations

The current study has several limitations. First, given that our aim was to account for qualitative differences in online health information seekers’ attitudes, technical skills, and cognitive strategies, we were able to observe only a small number of individuals, which, in turn, limited our ability to identify meaningful differences at a more fine-grained level. Thus, as an observational and qualitative study, our work should be followed up with controlled experiments and larger sample sizes. Second, the degree of distrust toward online information may be specific to German health information seekers given that Germany has a universal health care system, and visiting and asking his or her doctor questions usually does not incur any costs for the patient. Future studies should corroborate our findings related to attitudes toward online information in different cultures and health care systems. Third, we used demographic and Internet-specific variables to identify reliable differences in Web-use skill. However, age and educational differences between cohorts also imply differences in prior exposure to and understanding of health issues, which may have equally affected the observed distribution of search and evaluation strategies across cohorts (see Table 4). Future studies should clarify whether older Web users mainly search to confirm a priori opinions based on richer health experiences or to compensate for poor Web-use skills. Fourth, although we carefully designed the scenarios to match common search motivations (see Table 1), they may have seemed irrelevant to some of the participants. Thus, our findings may differ from online behavior motivated by real health concerns. Also, the researcher conducting study sessions was careful to let participants know they had all the time they wanted to search. However, we cannot preclude that her presence might have affected participants’ online behavior.

### Implications

Based on the findings, we summarized avenues for interventions in Table 5, which we elaborate in turn. To prevent inequalities in terms of data access, interventions should focus on improving people’s basic Web-use skills. A particular focus of these interventions should be skills related to search implementation and site selection efforts. To help people identify good-quality information independent of Web-use skills and simple stopping rules, search engine results should be visually labeled according to quality criteria.

**Table 5:** Avenues for interventions based on findings related to Web-use skill differences.

Finding	Intervention
Less-skilled health information seekers are concerned with managing data quantity.	Specify search terms and information categories to be searched for or use natural language phrases.
Less-skilled health information seekers pay little attention to information source when selecting websites.	Restrict search results to trusted sites by adding the command “site:” to queries.
Most health information seekers stop searching after finding a first piece of evidence satisfying search intentions, without cross-referencing.	Use multiple tabs in a browsing window to facilitate comparison of search engine results.
Web-use skills are not sufficient to guarantee access to and use of good-quality health information [27].	Visually label search engine results according to quality criteria.

#### Specify Search Words

Less-skilled users stated information overload as a major obstacle to using the Internet. Skilled Web users concatenated two or more search terms to specify the topic (eg, a medication) and the information category (eg, side effects) they were looking for. Specifying search intentions helped them constrain search results and avoid unrelated information. A similar effect was achieved by 2 less-skilled participants who entered whole natural language phrases instead of search terms (eg, “What are the side effects of eszoplicone?”). This intuitive strategy may be appropriate for users with low Web-use skills, helping them translate search intentions into targeted queries.

#### Restrict Search to Trusted Sites

Less-skilled participants paid little attention to information source when selecting websites. To avoid low-quality evidence, users may add the “site:” command to search terms (eg, “search term site: Netdoktor,” “search term site: NIH”), which yields results stemming exclusively from the identified website, circumventing the need to identify trustworthy sites from a plethora of options.

#### Use Multiple Tabs to Compare Findings

Neither cohort systematically cross-referenced online information. However, 2 of the skilled participants’ navigation strategies may provide a remedy. Moving from sequential to parallel browsing, they opened several websites they considered relevant, each in a new tab, and then compared contents. The parallel browsing function may facilitate cross-referencing behavior.

#### Visually Label Search Engine Results

Both cohorts used simple search and stopping rules. They consulted only a few links and stopped searching once they retrieved the first piece of information that was consistent with their search intentions. Thus, one way to facilitate access to good-quality information independent of Web-use skill level is to visually structure result rankings according to quality criteria. Although search engines may use quality criteria to rank order search results, it is currently difficult to assess which of the displayed search results link to good-quality information. In 2010, Google USA partnered with the US National Institutes of Health (NIH) to change this situation [28]. When users of Google.com search diseases or selected medications, the first and visually separated result links to an NIH-sponsored website. Alas, similar ventures do not yet exist for search algorithms in other countries. In Germany, for example, searching for brand name drugs on Google yields links to pharmaceutical companies, industry-sponsored websites, or advertising. A visual acknowledgment of the quality structure underlying search results assures that the first information that health information seekers come across and select is from high-quality sources. Given that results delivered by branded search engines are generally trusted [8], this increases the chances for Web users to find good-quality online information, independent of Web-use skills or the use of simple cognitive strategies.

## Abbreviations

MMR: measles-mumps-rubella
NIH: National Institutes of Health
PSA: prostate-specific antigen
